# Usefulness of comprehensive targeted multigene panel sequencing for neuromuscular disorders in Korean patients

**DOI:** 10.1002/mgg3.947

**Published:** 2019-09-01

**Authors:** Jihye Park, Hyun Mi Oh, Hye Jung Park, Ah‐Ra Cho, Dong‐Woo Lee, Ja‐Hyun Jang, Dae‐Hyun Jang

**Affiliations:** ^1^ Department of Rehabilitation Medicine, Eunpyeong St. Mary's Hospital, College of Medicine The Catholic University of Korea Seoul Republic of Korea; ^2^ Department of Rehabilitation Medicine, National Traffic Injury Rehabilitation Hospital, College of Medicine The Catholic University of Korea Seoul Republic of Korea; ^3^ Department of Rehabilitation Medicine, Incheon St. Mary’s Hospital College of Medicine The Catholic University of Korea Seoul Republic of Korea; ^4^ Green Cross Genome Yongin Republic of Korea

**Keywords:** Neuromuscular disorder, Next‐generation sequencing, Targeted multigene panel sequencing, Whole exome sequencing

## Abstract

**Background:**

Multigene panel sequencing (MGPS) is the first‐line option in diagnostic testing for genetically heterogeneous but clinically similar conditions, such as neuromuscular disorders (NMDs). In this study, we aimed to assess the utility of comprehensive NMD MGPS and the need for updated panels.

**Methods:**

All patients were analyzed by either of two versions of the NMD MGPS and by chromosomal microarray and karyotype testing. Four patients with negative NMD MGPS results underwent whole exome sequencing.

**Results:**

In total, 91 patients were enrolled, and a genetic diagnosis was made in 36 (39.6%); of these, 33 were diagnosed by the comprehensive NMD MGPS, two were confirmed by chromosomal microarray, and one was diagnosed by whole exome sequencing. For MGPS, the diagnostic yield of Version 2 (19/52; 36.5%) was a little higher than that of Version 1 (14/39; 35.9%), and one gene identified in Version 2 was not included in Version 1. A total of 36 definitive and nine possible causative variants were identified, of which 17 were novel.

**Conclusion:**

A more comprehensive panel for NMD MGPS can improve the diagnostic efficiency in genetic testing. The rapid discovery of new disease‐causing genes over recent years necessitates updates to existing gene panels.

## 1 INTRODUCTION

Neuromuscular disorder (NMD) is a broad diagnostic term that encompasses many diseases affecting the neuromuscular system. Next‐generation sequencing (NGS) is a routine diagnostic approach in molecular genetic diagnostic of NMDs due to their several characteristics including unspecific clinical findings, genetic and clinical heterogeneity, and yet unidentified genes (Savarese et al., 2016). Before NGS was developed, NMDs had routinely been diagnosed using a gene‐by‐gene approach, which was time consuming, expensive, and complex. In NMDs, one of large human genes such as TTN is mutated; therefore, it was difficult to be diagnosed (Tian et al., 2015). By applying massively parallel sequencing, NGS has dramatically reduced the time and cost associated with molecular diagnosis compared with the traditional approach (Mamanova et al., 2010; Rehm et al., 2013).

Recently, targeted multigene panel sequencing (MGPS), a NGS technique, has become widely used in molecular diagnosis. Targeted MGPS generally ensures that all coding exons of the gene of interest are targeted and that all exons have sufficiently deep coverage. Consequently, it is considered as a first‐line test for diagnosing inherited diseases like NMDs that are genetically heterogeneous but share similar clinical features (Volk & Kubisch, 2017). Targeted MGPS can perform comprehensive sequencing of most of associated genes with the disease, while allowing data analysis to focus on validated gene; therefore, it is advantageous in terms of cost and time (Walsh & Cook, 2017). However, it is limited by the fact that genes not included in the panel cannot be analyzed. We can therefore increase the sensitivity of testing and reduce the rate of undiagnosed cases by including newly found genes or genes with traditionally low mutation rates (Klee, Hoppman‐Chaney, & Ferber, 2011).

Another limitation of targeted MGPS in the diagnosis of NMDs is that the most of NGS panels have been focused on a certain NMD category, such as congenital myopathy, muscular dystrophy, motor neuron disease, hereditary spastic paraplegia (SPG), or hereditary polyneuropathy (D'Amore et al., 2018; Lim et al., 2011; Mamanova et al., 2010; Valencia et al., 2012; Vasli & Laporte, 2013; Wu, Brady, Shoffner, & Tarnopolsky, 2018). This means that clinicians must select a specific panel they consider to be most appropriate, despite many NMDs having overlapping phenotypes or clinical heterogeneity (Xue, Ankala, Wilcox, & Hegde, 2015). Silver syndrome, which is a complicated SPG, is a good example of a disease that is caused by a *BSCL2* mutation that can present with neurologic features on a spectrum between SPG and Charcot–Marie–Tooth (CMT) neuropathy. Hence, a patient with Silver syndrome may have an overlap of spasticity with hyperreflexia, muscle atrophy, foot deformity, and ataxia, making it difficult for clinicians to select a specific panel (Timmerman, Clowes, & Reid, 2013). Comprehensive MGPS containing all genes known to be involved in NMDs may be a more efficient diagnostic tool, yet only a few studies of comprehensive MGPS for NMDs have been conducted (Ankala et al., 2015; Tian et al., 2015). Therefore, we created a comprehensive MGPS panel for NMDs including myopathy, neuropathy, ataxia, spastic paraplegia, motor neuron disease, neuromuscular junction disorders, leukodystrophy, and related conditions (e.g. channelopathy). As the study progressed, we updated the panel, providing an opportunity to compare two versions.

In this study, we aimed to assess the usefulness of comprehensive MGPS testing for undiagnosed patients with suspected NMDs, and to investigate whether a benefit was gained from updating the panel based on more current data.

## 2 PATIENTS AND METHODS

### 2.1 Study design and participants

Patients were enrolled from tertiary hospitals between June 2016 and May 2018. An NMD specialist examined all patients with suspected NMDs of genetic etiology. When no diagnosis was identified by conventional genetic and laboratory testing, an additional genetic testing was performed. Patients with suspected fascioscapulohumeral muscular dystrophy were first subjected to pulse‐field gel electrophoresis and methylation analysis, and if negative, we performed MGPS test. Patients who met the following criteria were excluded: any history of cerebrovascular accident, head trauma, drug or toxic agent exposure, autoimmune disease or any suspected nonhereditary NMD or repeat expansion disease. Participants provided written informed consent according to the study protocol, which was reviewed and approved by our Institutional Review Board (IRB approval number: XC17ONDI0025).

All patients were analyzed by a comprehensive NMD MGPS test. Two versions of the test were used in this study: the initial panel (Version 1) and the optimized panel (Version 2). Patients who underwent genetic testing between June 2016 and September 2017 were analyzed using Version 1, while those tested between October 2017 and May 2018 were analyzed using Version 2. In all patients, we also performed chromosomal microarray and concurrent karyotyping as first‐line cytogenetic diagnostic tests. Whole exome sequencing (WES) was performed in patients when deemed necessary by clinicians and if consent was obtained. When available, the origin of the variants and imbalances were determined through parental studies.

### 2.2 Comprehensive NMD MGPS test version 1

MGPS Version 1 comprised the genes implicated in NMDs, as detailed in the Gene Table of Neuromuscular Disorders (Bonne, Rivier, & Hamroun, 2018; Kaplan & Hamroun, 2013), Genetics Home Reference (National Library of Medicine ), the ClinVar archives (National Center for Biotechnology Information ), Online Mendelian Inheritance in Man (OMIM) database (Johns Hopkins University ), and other articles on NMD panels (Ankala et al., 2015; Savarese et al., 2014; Tian et al., 2015). Information on the function of each gene was based on the contents of OMIM. Among the NMDs, repeat expansion diseases such as myotonic dystrophy (*DMPK* and *CNBP* gene) and mitochondrial diseases were excluded. MGPS Version 1 analyzed 293 genes (5,609 exons) associated with hereditary NMDs (Table S1). In this version, NMDs and their causative genes were categorized as myopathy (95 genes), motor neuron disease (16 genes), ataxia (76 genes), neuropathy (79 genes), neuromuscular junction disorder (11 genes), SPG (41 genes), leukodystrophy (11 genes), and other (22 genes).

### 2.3 Comprehensive NMD MGPS test version 2

When Version 1 of the panel was designed, lack of experience and an inadequate review process led to the construction of a gene panel that only combined commercially available panels. Subsequently, we created a taskforce consisting of clinical geneticists, molecular geneticists, and NMD specialists. This taskforce then performed a comprehensive review of the genes known to cause NMDs, including newly discovered ones. In addition, we excluded genetic movement disorders, such as those with a Huntington's disease phenotype. In this way, the comprehensive NMD MGPS Test Version 2 (MGPS Version 2) was created.

MGPS Version 2 analyzed 410 genes (7,154 exons) associated with hereditary NMDs (Table S2). From the 293 genes in Version 1, we deleted 28 and added 145 when creating Version 2. The final version comprised 136 genes related to myopathy, 40 related to motor neuron diseases, 91 related to ataxia, 106 related to neuropathy, 13 related to neuromuscular junction disorders, 54 related to SPG, 28 related to leukodystrophy, and 13 in the other category.

### 2.4 DNA samples and sequencing experiments

Genomic DNA was sampled from a patient's peripheral blood. Library preparation and target enrichment were performed by hybridization capture and custom oligo design, and synthesis was done by Celemics (Korea; MGPS Version 1) or Agilent (MGPS Version 2). Massively parallel sequencing was done using 2 × 150 bp in the paired‐end mode of MiSeq platform (Illumina, San Diego, CA). Sequence reads were trimmed with Trimmomatic (Version 0.33) and aligned with the Burrows–Wheeler Aligner (Version 0.7.12, MEM algorithm). Local realignment and recalibration were performed with the Genome Analysis Tool Kit (Version 3.5) (McKenna et al., 2010) after duplicated reads were eliminated with Picard (Version 1.96) (Broad Institute ). Variant calling was also performed using the Genome Analysis Tool Kit. Variants were annotated by Variant Effect Predictor (Version 88) (McLaren et al., 2016) and dbNSFP (Version 3.0) (Liu, Wu, Li, & Boerwinkle, 2016). Common variants with a minor allele frequency ≥ 1% were filtered out using public databases (i.e., 1,000 Genomes Project [European Bioinformatics Institute ]), Exome Variant Server (Washington University), ExAC browser (Exome Aggregation Consortium , and gnomAD browser (Genome Aggregation Database ). The average depth of coverage of MGPS Version 1 was 157× and 99% of target bases were covered by more than 10× sequence reads. The average coverage depth of MGPS Version 2 was 210× and 97% of target bases were covered by more than 10× sequence reads. All pathogenic variations were confirmed by conventional Sanger sequencing.

### 2.5 Array‐based comparative genomic hybridization

Genomic DNA was extracted from peripheral blood using the QIAamp DNA Mini Kit (Qiagen). The DNA was quantified spectrophotometrically using a ND‐1000 (Nanodrop Technologies). Array‐based comparative genomic hybridization analysis was then performed with a SurePrint G3 Human CGH Microarray 8x60K kit (Agilent Technologies), which consisted of 62,976 oligonucleotide probes spaced at 41 kbp intervals (median probe spacing) throughout the genome. Normal male or female DNA (Agilent Technologies) was used as controls. DNA digestion, labeling, and hybridization were performed according to the manufacturers' instructions. Scanned images were quantified using Agilent Feature Extraction software (v10.0), and the resulting data were imported into Agilent Genomic Workbench 7.0.4.0 software for visualization. Copy number variations were detected, using the Aberration Detection Method‐2 (ADM‐2) algorithm. Genomic positions were defined according to the GRCh37/hg19 Assembly of the Human Genome (February 2009).

### 2.6 Whole exome sequencing

Patients with negative results on the NMD MGPS underwent WES to identify genetic causes. To generate standard exome capture libraries, we used the Agilent SureSelect Target Enrichment protocol for the Illumina paired‐end sequencing library (ver. B.3, June 2015) with a 200 ng input of genomic DNA. In all cases, the SureSelect Human All Exon V5 probe set was used, before sequencing was performed using the HiSeq™ 2,500 platform (Illumina). Variant calling and annotation were performed in the same manner as for comprehensive NMD MGPS.

### 2.7 Variant interpretation

The pathogenicity of variants was classified as causative and possible causative, based essentially on the American College of Medical Genetics (ACMG) guideline (Richards et al., 2015). Pathogenic and likely pathogenic variants of the ACMG guideline were considered significant, and if the phenotypes and clinical tests were highly specific and/or the overall pattern of the pedigree was consistent with the genotypes, variants of uncertain significance were also considered significant. The causative and possible causative categories were defined based on the ACMG guideline, the correlation between phenotypes and genotypes, previous literature reports, pedigrees, and the immunohistologic, radiologic, and electrodiagnostic findings. If there was sufficient evidence, and the data were consistent, a “causative” label was given; if some evidence was missing, a “possible causative” label was given. All variants included in this article have been submitted to the ClinVar (http://www.ncbi.nlm.nih.gov).

## 3 RESULTS

### 3.1 Participants

A total of 91 patients (54 males and 37 females) were enrolled with a mean age of 30.72 ± 22.16 years (range 1–80 years). Among these, 39 were analyzed by MGPS Version 1 and 52 by MGPS Version 2; however, four patients with negative results by MGPS underwent WES. The mean age at symptom onset was 20.87 ± 21.25 years (range, 0–79 years).

**Figure 1:**
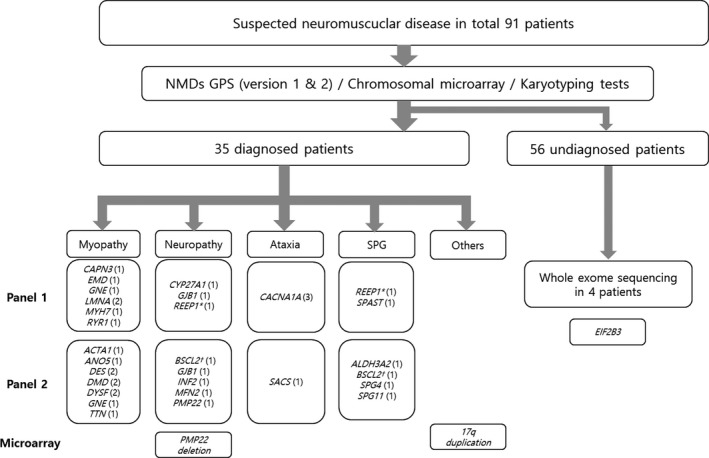
Flow chart for the analysis of patients with NMD in this study. ^*,†^ *REEP1* and *BSCL2* genes are associated with both neuropathy and SPG. MGPS, multi‐gene panel sequencing; NMD, neuromuscular disorder; SPG, spastic paraplegia

Figure 1 shows that the clinically suspected diagnoses were myopathy (35 patients), SPG (20 patients), neuropathy (15 patients), ataxia (12 patients), motor neuron disease (six patients), leukodystrophy (one patient), and others (two patients). A family history of NMD was reported in nine patients. Genetic causes were idendified in 36 of the 91 patients (39.6%), with 33 diagnosed by comprehensive NMD MGPS, two confirmed by chromosome microarray, and one diagnosed by WES (Figure 1).

### 3.2 Causative and possible causative variants

Among the 33 patients with genetic causes identified by NMD MGPS, 43 variants were identified and classified as either pathogenic (*n* = 31), likely pathogenic (*n* = 7), or of uncertain significance (*n* = 5) according to the ACMG guideline. These consisted of 22 missense, five splicing, eight frameshift, two in‐frame deletion, and six nonsense variants. Of note, 36 were considered definitive causative variants and seven were considered possible causative variants, with 15 identified as novel variants that had not previously been reported. Another two novel possible causative variants were identified by WES (Table 1).

**Table 1:** Summary of 34 patients diagnosed with causative/possible causative variants by MGPS and WES

Patient ID	Sex	Current age, years	Onset age, years	Gene	Panel version	Variants	Genotype	Family test	ACMG score	Phenotype	Causative	References	Novel
1	F	1.7	1.25	*EIF2B3*	WES	NM_020365.4: c.89T>C (p.Val30Ala) NM_020365.4: c.706C>G (p.Gln236Glu)	Heterozygote Heterozygote	Yes	VUS^‡^ VUS	Leukoencephalopathy with vanishing white matter	Possible Possible	This study This study	Yes Yes
26	F	33	NB	*MYH7*	1	NM_000257.3: c.1498_1500del (p.Glu500del)	Heterozygote	Yes	Likely pathogenic	Laing Distal Myopathy	Possible	This study	Yes
27	M	58	25	*REEP1*	1	NM_022912.2: c.603delC (p.*202Argfs*21)	Heterozygote	No	VUS	SPG31/HMN5B	Possible	This study	Yes
28	M	53	31	*SPG4*	1	NM_014946.3: c.1307C>T (p.Ser436Phe)	Heterozygote	No	Pathogenic	Spastic paraplegia 4	Causative	*Neurology* 2000;55:1388–90 (Hentati et al., 2000)	No
29	F	10	NB	*RYR1*	1	NM_000540.2: c.4496_4497delTT (p.Phe1499Cysfs*47) NM_000540.2: c.9716T>A (p.Met3239Lys)	Heterozygote Heterozygote	Yes	Pathogenic Likely pathogenic	Minicore myopathy with external ophthalmoplegia	Causative Causative	This study This study	Yes No
30	M	1.3	NB	*LMNA*	1	NM_005572.3: c.745C>T (p.Arg249Trp)	Heterozygote	No	Pathogenic	LMNA related congenital muscular dystrophy	Causative	*Ann Neurol* 2008;64:177–86 (Quijano‐Roy et al., 2008)	No
31	M	24	17	*CACNA1A*	1	NM_000068.3: c.3855C>G (p.Tyr1285*)	Heterozygote	Yes	Pathogenic	Episodic ataxia type 2	Causative	This study *Eur J Neurol* 2017;24(7):e43‐e44 (Lee, Jang, Jang, & Kim, 2017)	Yes
32	M	42	42	*CYP27A1*	1	NM_000784.3: c.1420C>T (p.Arg474Trp)	Homozygote	No	Pathogenic	Cerebrotendinous xanthomatosis	Causative	*J Lipid Res* 1994;35:1031–9 (K. S. Kim et al., 1994)	No
33	F	46	20	*LMNA*	1	NM_170707.3: c.1412G>C (p.Arg471Pro)	Heterozygote	No	VUS	LMNA related limb‐girdle muscular dystrophy	Possible causative	This study	Yes
34	M	32	20	*GNE*	1	NM_001128227.2: c.131G>C (p.Cys44Ser) NM_001128227.2: c.258−8G>A	Heterozygote Heterozygote	No	Pathogenic VUS	GNE myopathy	Causative Possible causative	*Hum Mutat* 2014;35:915–26 (Celeste et al., 2014) This study	No Yes
35	M	53	40	*GJB1*	1	NM_000166.5: c.590C>T (p.Ala197Val)	Hemizygote	No	Pathogenic	Charcot–Marie–Tooth Neuropathy X Type 1	Causative	*Clin Genet* 2012;81:142–9(Y. Kim et al., 2012)	No
36	M	55	20	*EMD*	1	NM_000117.2: c.101dupA (p.Tyr34*)	Hemizygote	No	Pathogenic	Emery–Dreifuss muscular dystrophy 1	Causative	*Neuromuscul Disord* 1999;9:159–65(Yates et al., 1999)	Yes
37	F	10	NB	*CACNA1A*	1	NM_001127221.1: c.4991G>A (p.Arg1664Gln)	Heterozygote	Yes	Pathogenic	Nonprogressive congenital cerebellar ataxia	Causative	*J Neurol Sci*. 2006;241(1–2):13–7(Tonelli et al., 2006)	No
38	F	23	15	*CACNA1A*	1	NM_001127221.1: c.5035C>T (p.Arg1679Cys)	Heterozygote	Yes	Pathogenic	Episodic ataxia, type 2	Causative	*J Neurol Sci* 2010;291(1–2):30–6(Mantuano et al., 2010)	No
39	F	45	25	*CAPN3*	1	NM_000070.2: c.1118G>A (p.Trp373*) NM_000070.2: c.1795dupA (p.Thr599Asnfs*33)	Heterozygote Heterozygote	No	Pathogenic Pathogenic	Muscular dystrophy, limb‐girdle, type 2A	Causative Causative	This study *Muscle Nerve* 1998;21:1493–501 (Kawai et al., 1998)	Yes No
72	M	35	35	*DYSF*	2	NM_003494.3: c.1284+2T>C NM_003494.3: c.5303G>A (p.Arg1768Gln)	Heterozygote Heterozygote	Yes	Pathogenic Likely pathogenic	Muscular dystrophy, limb‐girdle, type 2B	Causative Causative	*J Neurol Sci* 2003;211(1–2):23–8 (Tagawa et al., 2003) This study	No No
73	F	7	2	*SACS*	2	NM_014363.5: c.12973C>T (p.Arg4325*) NM_014363.5: c.11101T>C (p.Trp3701Arg)	Heterozygote Heterozygote	No	Likely pathogenic VUS	Autosomal recessive spastic ataxia of Charlevoix‐Saguenay	Causative Possible causative	*J Neurol *2006;253:1372–3 (Yamamoto et al., 2006) This study	No No
74	M	50	4	*MFN2*	2	NM_014874.3: c.1090C>T (p.Arg364Trp)	Heterozygote	No	Pathogenic	Charcot–Marie–Tooth disease, axonal, type 2A	Causative	*Neurology *2011;76:1690–6 (Feely et al., 2011)	No
75	M	14	8	*BSCL2*	2	NM_032667.6: c.269C>T (p.Ser90Leu)	Heterozygote	No	Pathogenic	HMN5A (Neuropathy, distal hereditary motor, type VA), SPG17 (spastic paraplegia−17)	Causative	*Muscle Nerve* 2007;36:384–6 (Cho, Sung, & Ki, 2007)	No
76	F	8	NB	*INF2*	2	NM_022489.3: c.311G>A (p.Cys104Tyr)	Heterozygote	No	Likely pathogenic	Charcot–Marie–Tooth disease, dominant intermediate E	Causative	*NEJM *2011;365:2377–88 (Boyer, Nevo, et al., 2011)	Yes
77	M	9	NB	*SPG4*	2	NM_014946.3: c.1253_1255del(p.Glu418del)	Heterozygote	Yes	Pathogenic	Spastic paraplegia 4	Causative	*J Neurol* 2013;260:906–909 (Guthrie et al., 2013)	No
78	F	17	NB	*PMP22*	2	NM_000304.3: c.281delG (p.Gly94Alafs*17)	Heterozygote	No	Pathogenic	Charcot–Marie–Tooth disease type 1E	Causative	*Muscle Nerve* 1997;20:1308–10 (Ionasescu et al., 1997)	No
79	F	37	7	*GNE*	2	NM_005476.5: c.2135T>C (p.Met712Thr)	Homozygote	Yes	Pathogenic	GNE myopathy	Causative	*Nat Genet* 2001;29:83–7 (Eisenberg et al., 2001)	No
80	M	23	20	*ANO5*	2	NM_213599.2: c.1158delT (p.Phe386Leufs*41) NM_213599.2: c.1640G>A (p.Arg547Gln)	Heterozygote	Yes	Pathogenic VUS	Miyoshi muscular dystrophy 3 or Muscular dystrophy, limb‐girdle, type 2L	Causative Possible causative	This study *Neuromuscul Disord* 2013;23:456–60 (van der Kooi et al., 2013)	Yes No
81	F	10	7	*TTN*	2	NM_133378.4: c.26231−1G>C NM_133378.4: c.85108dup (p.Arg28370Lysfs*15)	Heterozygote Heterozygote	Yes	Pathogenic Pathogenic	Muscular dystrophy, limb‐girdle, type 2J	Causative Causative	This study This study	Yes Yes
82	M	33	7	*DMD*	2	NM_004006.2: c.1652G>A (p.Trp551*)	Hemizygote	No	Pathogenic	Duchenne muscular dystrophy	Causative	This study	Yes
83	M	58	40	*DES*	2	NM_001927.3: c.1255C>T (p.Pro419Ser)	Heterozygote	No	Pathogenic	Myofibrillar myopathy	Causative	*Neuromuscul Disord* 2007;17:443–50 (Olive et al., 2007)	No
84	M	31	20	*SPG11*	2	NM_025137.3: c.3291+1G>T NM_025137.3: c.5410_5411del (p.Cys1804Profs*25)	Heterozygote Heterozygote	No	Pathogenic Pathogenic	Spastic paraplegia 11	Causative Causative	*J Neurol *2009;256:1714–8 (Kim et al., 2009) *J Neurol* 2009;256:1714–8 (Kim et al., 2009)	No
85	M	22	20	*DES*	2	NM_001927.3: c.1043A>C (p.Gln348Pro)	Heterozygote	No	Pathogenic	Myofibrillar myopathy	Causative	*PLoS One* 2014;9:e115470 (Fichna et al., 2014)	No
86	F	1.8	NB	*ALDH3A*	2	NM_000382.2: c.1291_1292del (p.Lys431Glufs*5) NM_000382.2: c.1309A>T (p.Lys437*)	Heterozygote Heterozygote	No	Pathogenic Pathogenic	Sjogren‐Larsson syndrome	Causative Causative	*Am J Hum Genet* 1999;65:1547–60 (Rizzo, Carney, & Lin, 1999) *J Child Neurol* 2013;28(10):1259–65 (Davis et al., 2013)	No No
87	F	55	25	*DYSF*	2	NM_003494.3: c.779C>G (p.Pro260Arg) NM_003494.3: c.2997G>T (p.Trp999Cys)	Heterozygote Heterozygote	Yes	Likely pathogenic Pathogenic	Muscular dystrophy, limb‐girdle, type 2B	Possible causative Causative	This study *Proc Jpn Acad *1999:75B;207–212 (Matsumura et al., 1999)	Yes No
88	M	3	2	*DMD*	2	NM_004006.2: c.9563+1G>A	Hemizygote	No	Pathogenic	Duchenne muscular dystrophy	Causative	This study	Yes
89	F	17	NB	*ACTA1*	2	NM_001100.3: c.739G>C (p.Gly247Arg)	Heterozygote	Yes	Likely pathogenic	ACTA1 gene related myopathy	Causative	This study	No
91	M	44	41	*GJB1*	2	NM_000166.5: c.394T>C (p.Trp132Arg)	Hemizygote	No	Pathogenic	Charcot–Marie–Tooth Neuropathy X Type 1	Causative	*Clin Genet* 2012:81:142–149(Kim et al., 2012)	No

Abbreviations: NB, new born; VUS, variant of uncertain significance.

**Figure 2:**
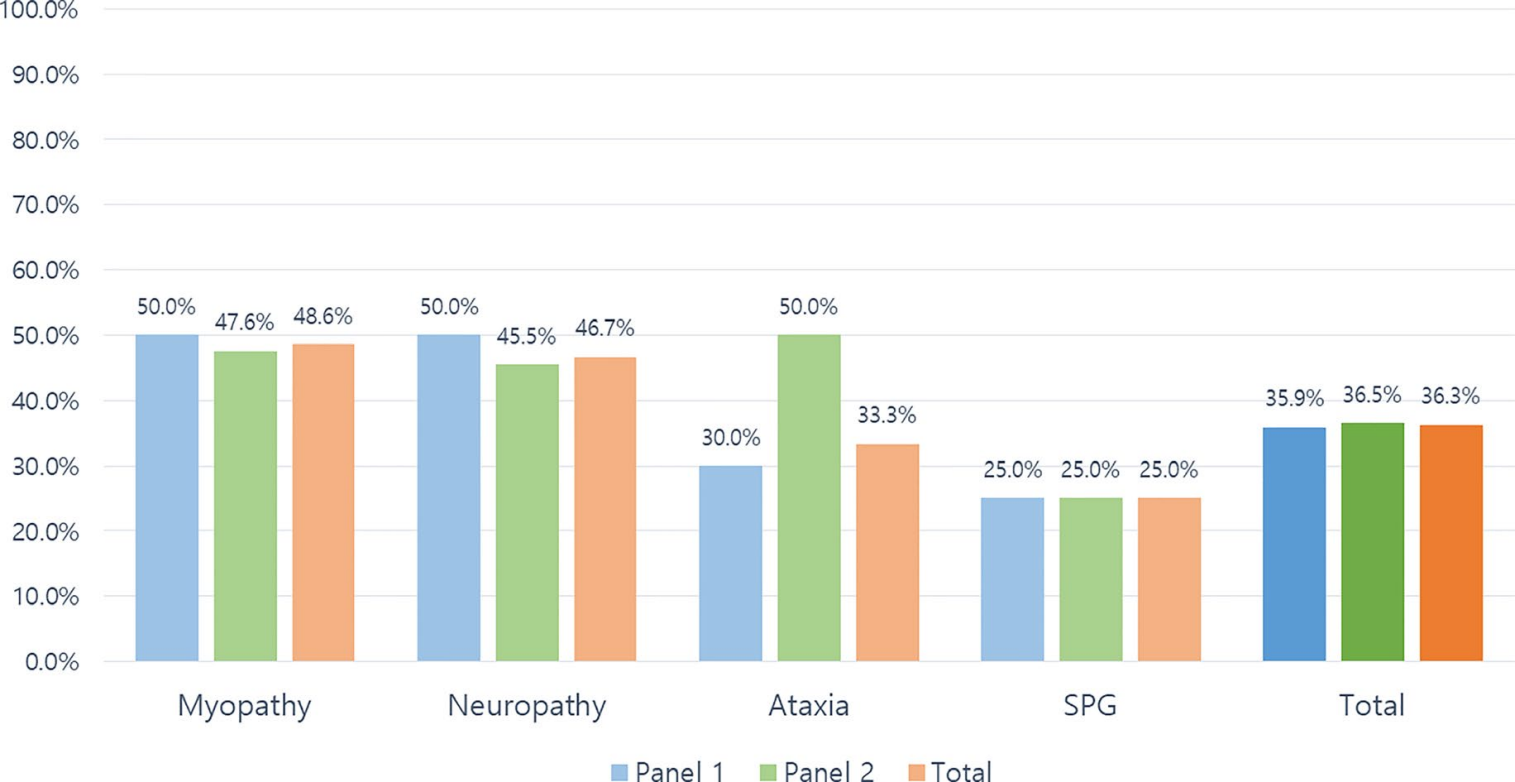
Diagnostic rate by neuromuscular disorder type and multi‐gene panel sequencing version

Diagnoses were made in 35.9% (14/39) and in 36.5% (19/52) of patients analyzed by MGPS Version 1 and MGPS Version 2, respectively (Figure 2). The diagnoses by disease categories (NMDs type) are shown in Figure 2; 48.6% (17/35) of patients with suspected myopathy were confirmed that diagnosis compared with 46.7% (7/15) for suspected neuropathy, 33.3% (4/12) for suspected ataxia, and 25.0% (5/20) for suspected SPG. In addition, there were two cases in which two different disease categories overlapped, such as neuropathy and SPG (*REEP1*, Patient ID 27; *BSCL2,* Patient ID 75). One patient who underwent WES was molecularly confirmed (*EIF2B3,* Patient ID 1). Finally, two patients were diagnosed by chromosomal microarray (*17q* duplication, Patient ID 18; *PMP22* deletion, Patient ID 69).

## 4 DISCUSSION

To date, there has been a little research into the use of comprehensive MGPS panels for NMD, with one study reporting a 60% diagnosis rate (Tian et al., 2015). However, the number of patients in that study was small (38 patients), most had myopathy, and the variants were broadly interpreted without using the ACMG guidance. By contrast, based on stricter criteria, the overall diagnosis rate of our panel was 36.3%, with diagnosis rates for myopathy and neuropathy of 48.6% and 46.7%, respectively. Whereas these data were broadly similar to those in previous reports, no cases of motor neuron disease were diagnosed in our very limited sample of just six patients. As was the case with previous studies (Chia, Chiò, & Traynor, 2018), the diagnosis rate was probably very low because most patients had no family history.

Presenting overlapping phenotypes is one of characteristics of NMDs (Xue et al., 2015). In this study, there were two cases of two overlapping disease categories. The first was a 58‐year‐old male (Patient ID 27) who underwent testing for suspected SPG, but who was finally diagnosed with spastic paraplegia 31 and distal hereditary motor neuronopathy type VB, caused by a mutation in *REEP1*. This gene is the third most common genetic cause of SPG (Zuchner et al., 2006), and it has been reported that a heterozygous splice site mutation can also cause autosomal dominant distal hereditary motor neuronopathy type VB (Beetz et al., 2008). The second was a 14‐year‐old male (Patient ID 75) who was tested for suspected CMT, but who was diagnosed with distal hereditary motor neuropathy type VA and spastic paraplegia 17 due to a mutation of *BSCL2*. It has been shown that Silver syndrome and some forms of hereditary motor neuronopathy result from a mutation of the same gene (Windpassinger, Wagner, Petek, Fischer, & Auer‐Grumbach, 2003). Similar cases of patients with phenotypic overlap between Silver syndrome and distal hereditary motor neuronopathy type V have also been reported (Brusse et al., 2009; van de Warrenburg et al., 2006).

One the other hand, clinical heterogeneity is another characteristic of NMDs (Vasli & Laporte, 2013). For example, mutations in *LMNA* (Patient ID 30 and 33) have been associated with many different phenotypes, including Emery–Dreifuss muscular dystrophy, Dunnigan‐type familial partial lipodystrophy, a form of dilated cardiomyopathy with conduction system disease, a form of familial partial lipodystrophy, limb‐girdle muscular dystrophy type 1B, a form of autosomal recessive axonal neuropathy (CMT), Hutchinson‐Gilford progeria syndrome, and mandibuloacral dysplasia (Mercuri et al., 2004). As with *LMNA* mutations, patients can have symptoms overlap and can present with a diverse range of clinical manifestations of different levels of severities when the same mutation is present. Therefore, given that it is often difficult for clinicians to select the best category for direct molecular diagnosis, a comprehensive MGPS test should be more appropriate for diagnosing NMD.

All genes in the patients diagnosed by MGPS Version 1 were included in Version 2, but one gene with a variant identified by Version 2 was not included in Version 1. This applied to an eight‐year‐old female with developmental delay from birth (Patient ID 76). The MGPS revealed a novel variant of *INF2*, which was diagnosed as an intermediate form of CMT. Variants in the *INF2* are known to cause focal segmental glomerulosclerosis and account for about 12%–17% of autosomal dominant cases (Boyer, Benoit, et al., 2011; Brown et al., 2010). In 2011, Boyer et al. identified that *INF2* mutations were a major cause of CMT associated with focal segmental glomerulosclerosis, accounting for 12 of 16 cases in their cohort (Boyer, Nevo, et al., 2011). When MGPS Version 1 was constructed, *INF2* was excluded because the gene mutation was considered insufficient to establish a diagnosis of CMT. However, *INF2* variants in CMT have been reported by several investigators since Boyer's first report, and at the time of producing Version 2 of the panel, inclusion of the gene was deemed appropriate. Thus, as new genes associated with NMDs are discovered over time, and as confirmatory studies are reported, panels must be updated.

**Figure 3:**
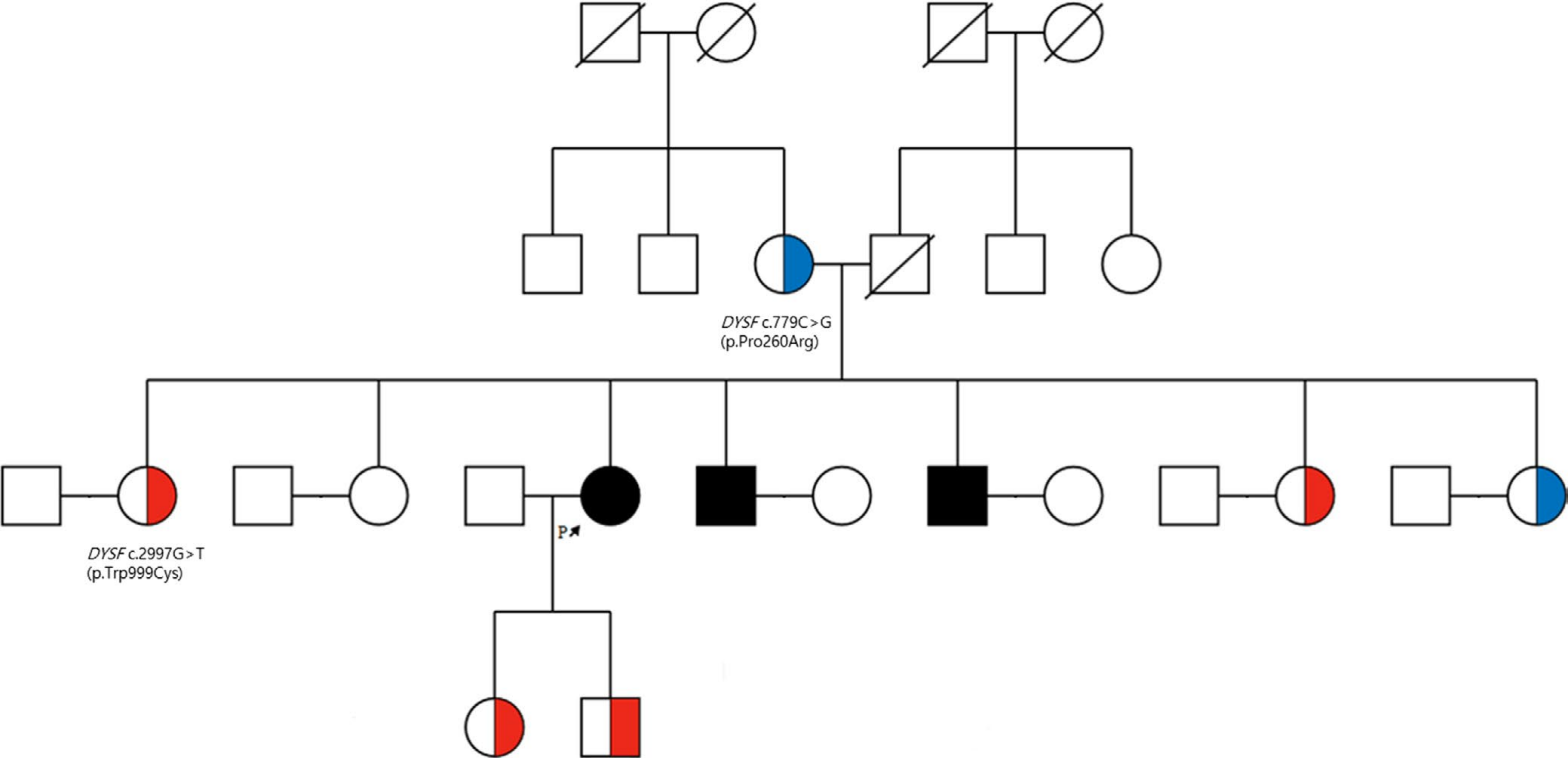
Family pedigree diagnosed with *DYSF* variants (Patient ID 87). The black filled‐in pedigree members are the patients (c.779C>G and c.2997G>T), and the blue half‐filled one indicates the heterozygous carrier (c.779C>G) while the red half‐filled is the heterozygous carrier (c.2997G>T)

Among the cases of NMDs diagnosed by MGPS and WES, 17 novel variants were identified as causative or possible causative that have not previously been reported (Table 1). Pathogenicity was confirmed for some cases through family testing. For example, a 55‐year‐old woman (Patient ID 87) who gradually developed proximal muscle weakness at the age of 25 years underwent testing with MGPS Version 2 for suspected myopathy. Two missense variants in *DYSF* were found (c.779C>G and c.2997G>T), and based on this, the patient was diagnosed with muscular dystrophy, limb‐girdle type 2B. The c.2997G>T variant has previously been reported to be pathogenic (Matsumura et al., 1999), but the c.779C>G variant was novel, and its association with disease has not previously been reported. Verifying the pathogenicity of this variant required intrafamilial segregation analysis and a muscle biopsy test. Although the patient refused to undergo muscle biopsy test (nonspecific myopathy had been identified 20 years previously), family testing of her mother confirmed co‐segregation (Figure 3). The novel c.779C>G variant was classified as a variant of unknown significance by the ACMG guideline (Richards et al., 2015), based on the evidence of not being found in the population database and the deleterious in‐silico predictions. However, the additional family study revealed that it was present in *trans* form and that the same two variants were present in siblings. Thus, this case could be reclassified as a likely pathogenic variant.

Five variants were judged to be of unknown significance according to the ACMG guideline, but these were also determined to be possible causative variants based on clinical symptoms. Three patients (Patient ID 1, 27, and 33) had clinical symptoms that were significantly associated with these genes. In another case, although clinical symptoms were related, parental and family tests could not be performed because the child was adopted (Patient ID 73). In three cases (Patient ID 27, 33, and 80), the clinical symptoms were associated with the gene but we were unable to perform parental or family tests because of special circumstances (e.g., their parents had died or they were an only child). Therefore, we concluded that these five variants of unknown significance were possibly causative.

One patient (Patient ID 1) who tested negative by MGPS had their diagnosis confirmed by WES. The mutated gene was *EIF2B3,* which has been identified in some patients with leukoencephalopathy with vanishing white matter, an autosomal recessive disorder. There have been few reports of Asian patients with this leukoencephalopathy caused by a mutation in *EIF2B3*. Therefore, this gene was missed because we only included Asian cases and did not include the gene in either version of the panel for MGPS, which may be considered a limitation of our approach. This indicates a need to update the test panel with newly found genes to ensure that sensitivity remains high. Thus, considering WES in patients with negative MGPS results would appear to be prudent.

As mentioned earlier, we used two versions of a comprehensive panel of NMD‐associated genes for MGPS. Version 1 of the panel consisted of 293 genes, whereas Version 2 consisted of 410 genes after removing unnecessary genes and adding newly discovered ones. In 2014, the cost of producing a panel with a large number of genes was prohibitive, and the lack of suitable reference databases and clinician experience meant that the panel only contained a small number of genes. However, we overcame these shortcomings and later produced an updated version during the course of our research; the resulting ability to compare both panels is an important strength of our study. A limitation, however, is that only four of 55 patients with negative results underwent WES. During the study, only a few patients agreed to perform further testing by WES; although some of these patients had died, most simply did not want further evaluation after receiving negative results by MGPS.

## 5 CONCLUSIONS

The findings of this study suggest that comprehensive MGPS for NMD can improve the efficiency of genetic diagnosis. As new causative genes are discovered, the gene panel will need to be updated.

## CONFLICT OF INTEREST

The authors have no conflict of interest to disclose and there was no financial support.
