# Supplementary material for: Usefulness of comprehensive targeted multigene panel sequencing for neuromuscular disorders in Korean patients
Source: Mol Genet Genomic Med. 2019 Sep 1;7(10):e00947. doi: 10.1002/mgg3.947 (PMC6785438; doi:10.1002/mgg3.947)
Supplement: Supplementary file 1 [file MGG3-7-e00947-s001.docx]

**Supplementary Table S1.** The NMDs-related genes in comprehensive MGPS Version 1.

| **No**. | **Category (numbers)** | **Genes** |
| --- | --- | --- |
| 1 | Myopathy (95) | *ACAD9, ACADL, ACADM, ACTA1, AGL, AMPD1, ANO5, ATP2A1, BAG3, BIN1, CAPN3, CAV3, CAVIN1, CCDC78, CFL2, CLCN1, CNTN1, COL6A1, COL6A2, COL6A3, CPT1B, CPT2, CRYAB, DAG1, DES, DMD, DNAJB6,* ***DNM2****, DYSF, EMD, FHL1, FKRP, FKTN, FLNC, GAA, GNE, GYS1, HADHA, HADHB, HSPG2, ISPD, ITGA7, KBTBD13, KLHL40, KLHL41, LAMA2, LARGE1, LDB3,* ***LMNA****, LPIN1,* ***MATR3****, MTM1, MTMR14, MYF6, MYH2, MYH7, MYOT, NEB, OPA1, OPA3, PABPN1, PFKM, PGAM2, PHKA1,* ***PLEC****,* ***POLG****, POLG2, POMGNT1, POMT1, POMT2, PYGM, RRM2B, RYR1,* ***SCN4A,*** *SELENON, SGCA, SGCB, SGCD, SGCG,* ***SIL1****, STAC3,* ***SUCLA2****,* ***SYNE1,*** *SYNE2, TCAP, TK2, TNNI2, TNNT1, TPM2, TPM3, TRIM32, TTN,* ***VCP****,* ***VPS13A****,* ***XK*** |
| 2 | Motor neuron disease (16) | ***ATP7A,*** *DCTN1,* ***DNAJB2****,* ***DYNC1H1****,* ***FIG4****, FUS, GLE1,* ***IGHMBP2****,* ***MATR3****,* ***PLEKHG5****,* ***SETX****, SMN1, SOD1,* ***SPG11****,* ***TRPV4,*** ***VCP*** |
| 3 | Ataxia (76) | *ABCB7,* ***ABHD12,*** *ACO2, AFG3L2, ANO10, APTX, ATCAY,* ***ATM****, ATP8A2, BEAN1,* ***C10orf2****,* ***CACNA1A****,* ***CACNB4****, CCDC88C,* ***CLCN2,*** *COQ8A, CWF19L1,* ***DNMT1****, EEF2, ELOVL4, ELOVL5, FGF14,* ***FLVCR1****, FXN, GOSR2, GRID2, GRM1, ITPR1,* ***KCNA1****, KCNC3, KCND3, KCNJ10, KIF1C, LAMA1, MARS2, MRE11, MTPAP, MTTP,* ***NOP56****, OPHN1, PDYN,* ***PEX7****,* ***PHYH****,* ***PLP1****,* ***PNKP****,* ***PNPLA6****,* ***POLG****, PRKCG,* ***PRPS1****,* |
| **No**. | **Category (numbers)** | **Genes** |
|  |  | *PTF1A, RNF216, RUBCN,* ***SACS****,* ***SETX****,* ***SIL1****, SLC1A3, SLC52A2, SLC9A1, SNX14, SPTBN2, STUB1,* ***SYNE1****, SYT14, TBP,* ***TDP1****, TGM6, TMEM240, TPP1, TTBK2,* ***TTPA****, VAMP1, VLDLR,* ***VRK1****,* ***WFS1****, WWOX, ZNF592* |
| 4 | Neuropathy (79) | *AARS,* ***ABHD12****, AIFM1,* ***ATL1****,* ***ATM****,* ***BSCL2****,* ***C10orf2****, CTDP1,* ***CYP27A1****,* ***DNAJB2****,* ***DNM2****,* ***DNMT1****,* ***DYNC1H1****, EGR2, ELP1, FGD4,* ***FIG4****,* ***FLVCR1****, GAN, GARS, GDAP1, GJB1, GLA, GNB4, HINT1, HOXD10, HSPB1, HSPB8,* ***IGHMBP2****,* ***KIF1A****, KIF1B, LITAF,* ***LMNA****, LRSAM1, MARS, MED25, MFN2, MPZ, MTMR2, NDRG1, NEFL, NGF,* ***NOP56****, NTRK1, PDK3,* ***PEX7****,* ***PHYH****,* ***PLEKHG5****,* ***PLP1****, PMM2, PMP22,* ***PNKP****,* ***PNPLA6****,* ***POLG****,* ***PRPS1****, PRX, RAB7A,* ***REEP1****, RETREG1,* ***SACS****, SBF2, SCN9A, SH3TC2, SLC12A6,* ***SPG11****, SPTLC1, SPTLC2,* ***TDP1****,* ***TRPV4,*** ***TTPA****, TTR, TYMP,* ***VPS13A****,* ***VRK1****,* ***WFS1****, WNK1,* ***XK****, YARS,* ***VCP*** |
| 5 | Neuromuscular junction disorder (11) | *CHAT, CHRNA1, CHRNB1, CHRND, CHRNE, COLQ, DOK7, MUSK,* ***PLEC****, RAPSN,* ***SCN4A*** |
| 6 | Spastic paraplegia (41) | *ALDH3A2, AP4B1, AP4E1, AP4M1, AP4S1, AP5Z1,* ***ATL1****,* ***BSCL2,*** *C12orf65, C19orf12, CYP2U1, CYP7B1, DDHD1, DDHD2, ERLIN2, FA2H, GAD1,* ***GALC****, GBA2,* ***GJC2, HSPD1****,* ***KIF1A****, KIF5A, L1CAM, NIPA1,* ***PLP1****,* ***PNPLA6****,* ***REEP1****, RTN2, SLC16A2,* *SLC33A1, SPAST,* ***SPG11****, SPART, SPG21, SPG7, TECPR2,* |
| **No**. | **Category (numbers)** | **Genes** |
|  |  | *VPS37A, WASHC5, ZFYVE26, ZFYVE27* |
| 7 | Leukodystrophy (11) | *ABCD1, ARSA,* ***CASK****,* ***CLCN2****, CLN5,* ***CYP27A1****,* ***GALC****,* ***GJC2****,* ***HSPD1****,* ***SUCLA2****, TUBB4A* |
| 8 | Channelopathy and others (22) | ***ATP7A****, ATP7B,* ***CACNA1A****, CACNA1S,* ***CACNB4****,* ***CASK****, CHRNG, ETFA, ETFB, FRMD7, GPR143, JPH3,* ***KCNA1****, KCNE3, KCNJ18, PANK2, RYR2,* ***SCN4A,*** *SGCE, SLC39A4, SLC9A6,* ***VPS13A*** |

A total of 293 genes responsible for the neuromuscular disorders are listed as 8 categories of diseases, and the number of genes is in parentheses. Repeated genes are shown in boldface type.
