# Supplementary material for: Usefulness of comprehensive targeted multigene panel sequencing for neuromuscular disorders in Korean patients
Source: Mol Genet Genomic Med. 2019 Sep 1;7(10):e00947. doi: 10.1002/mgg3.947 (PMC6785438; doi:10.1002/mgg3.947)
Supplement: Supplementary file 2 [file MGG3-7-e00947-s002.docx]

**Supplementary Table S2.** The NMDs-related genes in comprehensive MGPS Version 2.

| **No**. | **Category (numbers)** | **Genes** |
| --- | --- | --- |
| 1 | Myopathy (136) | *ACAD9, ACTA1, AGL, AMPD1, ANO5, ATP2A1, B3GALNT2, B4GAT1, BAG3, BIN1, CAPN3, CASQ1, CAV3, CAVIN1, CCDC78, CFL2,* ***CHCHD10,*** *CHKB, CLCN1, CNTN1, COL12A1, COL6A1, COL6A2, COL6A3, CPT2, CRYAB, DAG1, DES, DMD, DNAJB6,* ***DNM2,*** ***DPM1, DPM2, DPM3,*** *DYSF, EMD, FHL1, FKRP, FKTN, FLNC, GAA, GBE1, GMPPB, GNE, GYG1, GYS1, HADHA, HADHB,* ***HNRNPA1,*** *HNRNPA2B1, HNRNPDL, HRAS, HSPG2, ISCU, ISPD, ITGA7, KBTBD13, KLHL40, KLHL41, KLHL9, LAMA2, LAMP2, LARGE1, LDB3, LDHA, LIMS2,* ***LMNA,*** *LMOD3,* ***MATR3,*** *MEGF10, MICU1, MSTN, MTM1, MTMR14, MYF6, MYH2, MYH7,* ***MYH14,*** *MYO18B, MYOT, MYPN, NEB, PABPN1, PFKM, PGAM2, PGK1, PGM1, PHKA1, PLEC, PNPLA2,* ***POLG,*** *POLG2, POMGNT1, POMGNT2, POMK, POMT1, POMT2, PUS1, PYGM, RBCK1, RRM2B, RYR1,* ***SCN4A,*** *SELENON, SGCA, SGCB, SGCD, SGCG,* ***SIL1,*** *SLC22A5, SLC25A20, SMCHD1, SPEG,* ***SQSTM1,*** *STAC3, STIM1,* ***SUCLA2****,* ***SYNE1,*** *SYNE2, TCAP, TK2, TMEM43, TMEM5, TNNI2, TNNT1, TNPO3, TPM2, TPM3, TRAPPC11, TRIM32,* ***TRIP4,*** *TTN, TWNK,* ***VCP*** *, VMA21,* ***XK*** |
| 2 | Motor neuron disease (40) | ***ALS2****, ANG, ASAH1, ASCC1****, ATP7A,*** *BICD2,* ***CHCHD10,*** *CHMP2B, DCTN1,* ***DNAJB2, DYNC1H1,*** *ERBB3, FBXO3,* ***FIG4,*** *FUS, GLE1,* ***HNRNPA1, IGHMBP2,*** ***MATR3, NEFH,*** *OPTN, PFN1, PIP5K1C,* |
| **No**. | **Category (numbers)** | **Genes** |
|  |  | ***PLEKHG5,*** *PRPH,* ***SETX****, SIGMAR1,* ***SLC52A3,*** *SMN1, SOD1,* ***SPG11, SQSTM1,*** *TARDBP,* ***TRIP4, TRPV4,*** *UBA1, UBQLN2, VAPB,* ***VCP*** |
| 3 | Ataxia (91) | *ABCB7,* ***ABHD12,*** *ACO2, AFG3L2, ANO10, APTX, ATCAY,* ***ATM,*** *ATP1A3, ATP2B3, ATP8A2, BEAN1,* ***C10orf2,*** *CA8,* ***CACNA1A*** *, CACNA1G,* ***CACNB4,*** *CAMTA1, CCDC88C,* ***CLCN2,*** *COQ2, COQ8A, CWF19L1, DNAJC19,* ***DNMT1,*** *EEF2, ELOVL4, ELOVL5, FGF14,* ***FLVCR1,*** *FXN, GOSR2, GRID2, GRM1, ITPR1,* ***KCNA1,*** *KCNC3, KCND3, KCNJ10, KIF1C, LAMA1, MARS2,* ***MME****, MRE11, MTPAP, MTTP,* ***NOP56,*** *OPHN1, PDYN,* ***PEX7****,* ***PHYH****, PIK3R5,* ***PLP1****, PMPCA,* ***PNKP****,* ***PNPLA6****,* ***POLG****, PRICKLE1, PRKCG,* ***PRPS1****, PTF1A, RNF216, RUBCN,* ***SACS****,* ***SETX****,* ***SIL1****, SLC1A3, SLC52A2,* ***SLC52A3****, SLC9A1, SNX14, SPTBN2, STUB1,* ***SURF1****,* ***SYNE1****, SYT14,* ***TDP1****, TGM6, TMEM240, TPP1, TRPC3, TTBK2,* ***TTPA****, VAMP1, VLDLR,* ***VRK1****, VWA3B, WDR73,* ***WFS1****, WWOX, ZNF592* |
| 4 | Neuropathy (106) | *AARS,* ***ABHD12,*** *AIFM1, ARHGEF10,* ***ATL1,*** *ATL3,* ***ATM, BSCL2, C10orf2,*** *CCT5, COX6A1, CTDP1,* ***CYP27A1,*** *DCAF8, DHTKD1,* ***DNAJB2, DNM2, DNMT1,*** *DST,* ***DYNC1H1,*** *EGR2, ELP1,* ***FIG4,*** *FBLN5, FGD4,* ***FLVCR1,*** *GAN, GARS, GDAP1, GJB1, GJB3, GLA, GNB4, HARS, HINT1, HK1, HMBS, HOXD10,* |
| **No**. | **Category (numbers)** | **Genes** |
|  |  | *HSPB1, HSPB3, HSPB8,* ***IGHMBP2,*** *INF2, JPH1, KARS,* ***KIF1A,*** *KIF1B, LITAF,* ***LMNA****, LRSAM1, MARS, MED25, MFN2,* ***MME****, MPZ, MTMR2,* ***MYH14,*** *NDRG1,* ***NEFH,*** *NEFL, NGF,* ***NOP56,***  *NTRK1, PDK3,* ***PEX7, PHYH****,* ***PLEKHG5,*** ***PLP1,*** *PMM2, PMP22,* ***PNKP, PNPLA6, POLG, PRPS1,*** *PRX, RAB7A,* ***REEP1,*** *RETREG1,* ***SACS,*** *SBF1, SBF2, SCN10A, SCN11A, SCN9A, SEPT9, SH3TC2,* ***SLC5A7,*** *SLC12A6, SOX10,* ***SPG11,*** *SPTLC1, SPTLC2,* ***SURF1, TDP1, TFG,*** *TRIM2,* ***TRPV4, TTPA,*** *TTR, TYMP,* ***VCP, VRK1, WFS1,*** *WNK1,* ***XK,*** *YARS* |
| 5 | Neuromuscular junction disorder (13) | *AGRN, ALG14, ALG2, DPAGT1, GFPT1, LAMB2, MUSK, PREPL,* ***PLEC,*** *RAPSN,* ***SCN4A, SLC5A7,*** *SYT2* |
| 6 | Spastic paraplegia (54) | *ALDH18A1, ALDH3A2,* ***ALS2,*** *AMPD2, AP4B1, AP4E1, AP4M1, AP4S1, AP5Z1, ARL6IP1,* ***ATL1,*** *B4GALNT1,* ***BSCL2,*** *C12orf65, C19orf12, CAPN1, CYP2U1, CYP7B1, DDHD1, DDHD2, ENTPD1, ERLIN1, ERLIN2, FA2H, FARS2, GAD1,* ***GALC****, GBA2,* ***GJC2, HSPD1,*** *IBA57,* ***KIF1A,*** *KIF5A, L1CAM, MAG, NIPA1, NT5C2,* ***PLP1, PNPLA6, REEP1,*** *RTN2, SLC16A2, SLC33A1, SPAST,* ***SPG11****, SPART, SPG21, SPG7, TECPR2,* ***TFG****, VPS37A, WASHC5, ZFYVE26, ZFYVE27* |
| **No**. | **Category (numbers)** | **Genes** |
| 7 | Leukodystrophy (28) | *ABCD1, ADAR, ARSA, ASPA, ATP13A2,* ***CLCN2,*** *CSF1R, CTSD,* ***CYP27A1, GALC,*** *GFAP,* ***GJC2,*** *HEPACAM(MLC2A,2B),* ***HSPD1,*** *IFIH1, MFSD8, MLC1, POLR1C, POLR3A, POLR3B, PPT1, RNASEH2A, RNASEH2B, RNASEH2C, SAMHD1,* ***SUCLA2****, TREX1, TUBB4A* |
| 8 | Channelopathy and others (13) | ***ATP7A****, ATP7B,* ***CACNA1A,*** *CACNA1S,* ***CACNB4****, CHRNG,* ***DPM1, DPM2, DPM3, KCNA1,*** *KCNJ18, KCNJ2,* ***SCN4A*** |

A total of 410 genes responsible for the neuromuscular disorders are listed as 8 categories of diseases, and the number of genes is in parentheses. Repeated genes are shown in boldface type.
